# Cure or control: complying with biomedical regime of diabetes in Cameroon

**DOI:** 10.1186/1472-6963-8-43

**Published:** 2008-02-25

**Authors:** Paschal Kum Awah, Nigel Unwin, Peter Phillimore

**Affiliations:** 1 Institute of Health and Society, Faculty of Medical Sciences, University of Newcastle upon Tyne, NE2 4HH, UK; 2 Health of Populations in Transition (HoPiT) Research Group, PO Box 8046, Yaounde, Cameroon; 3 School of Geography, Politics and Sociology, University of Newcastle upon Tyne, NE2 4HH, UK

## Abstract

**Background:**

The objective of the study was to explore the cultural aspect of compliance, its underlying principles and how these cultural aspects can be used to improve patient centred care for diabetes in Cameroon.

**Methods:**

We used participant observation to collect data from a rural and an urban health district of Cameroon from June 2001 to June 2003. Patients were studied in their natural settings through daily interactions with them. The analysis was inductive and a continuous process from the early stages of fieldwork.

**Results:**

The ethnography revealed a lack of basic knowledge about diabetes and diabetes risk factors amongst people with diabetes. The issue of compliance was identified as one of the main themes in the process of treating diabetes. Compliance emerged as part of the discourse of healthcare providers in clinics and filtered into the daily discourses of people with diabetes. The clinical encounters offered treatment packages that were socially inappropriate therefore rejected or modified for most of the time by people with diabetes. Compliance to biomedical therapy suffered a setback for four main reasons: dealing with competing regimes of treatment; coming to terms with biomedical treatment of diabetes; the cost of biomedical therapy; and the impact of AIDS on accepting weight loss as a lifestyle measure in prescription packages. People with diabetes had fears about and negative opinions of accepting certain prescriptions that they thought could interfere with their accustomed social image especially that which had to do with bridging their relationship with ancestors and losing weight in the era of HIV/AIDS.

**Conclusion:**

The cultural pressures on patients are responsible for patients' partial acceptance of and adherence to prescriptions. Understanding the self-image of patients and their background cultures are vital ingredients to improve diabetes care in low-income countries of Sub-Sahara Africa like Cameroon.

## Background

In the last two decades, diabetes mellitus has emerged as a major public health problem in Sub-Sahara Africa. Quite recently, epidemiological studies [1,2] have revealed that diabetes and diabetes risk factors have contributed to the high mortality and morbidity of many adults [2-5]. Public health studies support the fact that good treatment outcomes have been thwarted by patients' unwillingness to comply with and adhere to treatment [6,7]. It has been illustrated in some countries that fewer than 80% of people living with diabetes take their medication as prescribed [2].

Compliance with appropriate, recommended, and prescribed health treatments simply means that a person is following a doctor's orders. Compliance is more likely when there is agreement and confidence regarding the medical diagnosis and prognosis [8]. Compliance is complicated by uncertainty about the nature of an illness and/or the effects of certain treatments, particularly medications. In everyday usage, the term compliance means deference and obedience, elevating the authority of medical expertise. Alternatively, adherence to medical advice refers to a somewhat more informed and equitable decision by a patient to stick with appropriate medical treatment. Concordance is an equal therapeutic agreement on treatment between the provider and a patient [6,9]. In any case, diabetes care cannot be effective or even evaluated if a patient does not follow a doctor's orders.

A diabetes treatment that is effective for one patient may not be beneficial for another patient, and diagnoses may evolve over time, complicating the issue of compliance. From a health provider's viewpoint, in order for effective medical treatments to have their desired effects, complying with treatments is absolutely necessary. The concept of medication management reflects this idea that the provider is responsible and in control, while the consumer is a docile body who is incapacitated by disease or condition [6,8]. From the perspective of patients, adherence to medical treatment is enhanced when there is a good health care relationship and when consumers openly share their health beliefs and experience of illness with their provider. Non-compliance can represent a significant risk and cost to the medical system. For providers, partial compliance or discontinuation of medications represents the difficulty of maintaining treatment successes over time [9,10]. Problems with compliance are often attributed to the patient, but may also reflect the appropriateness of a medication or treatment.

Most of the studies conducted to document the factors of non-compliance have focussed on western settings where diabetes care has a longer history [9,10]. These studies have laid emphasis on the biomedical therapies of diabetes, neglecting the cultural issues surrounding the care of diabetes, which is most important in sub-Sahara Africa where the interaction of dual medical frameworks co-exists. Though some studies [7,11] suggest that patients' non-compliance could be associated with reservations about drugs and lack of necessary knowledge on which to build an understanding of the condition and treatment, it is not fully understood if patients really perceive this as non-compliance. Though emphasis is being laid on adherence today, rather than compliance, many African health care providers still manage healthcare within the premise of compliance. This prompted the exploration of the cultural aspect of compliance, its underlying principles and how these can be used to improve patient centred care [12] for diabetes care in Cameroon.

## Methods

### Setting

The settings of the study were Bafut (a rural health district) and Biyem-Assi (an urban health district in Yaounde, the capital city) in Cameroon. Bafut is located 450 kilometres from Yaounde in the Northwest of Cameroon. Yaounde and Bafut have populations of about 1.8 million (455 433 inhabitants live in Biyem-Assi Health District) and 80 000 inhabitants respectively [13]. The two settings were chosen for fieldwork because of the existence of diabetes clinics at primary care level and a catchment of known diabetes patients. Table 1 presents general background characteristics of the study settings.

**Table 1:** General background characteristics of study settings at time of fieldwork

Characteristics of study settings	Urban Health District (Biyem-Assi Yaounde)	Rural Health District (Bafut)	Total
Population	453 833	80 000	
Age-adjusted prevalence of diabetes	5%	1%	Na
Number of health units	21	8	28
Number of health care providers	140	30	170
Number of diabetes clinics	4	4	8
Number of patients in initial sample	10	10	20
Number of families in initial sample	10	10	20
Number of healthcare workers trained in diabetes care	15	10	25
Frequency of consultations per patient	Monthly*	Monthly*	Na
Total number of patients consulting in sampled clinics at recruitment	150	55	205

* Monthly was the typical pattern, but not invariable.

The study covered a period of 25 months from June 2001 to June 2003. This study took a comparative anthropological approach because, firstly, it is the most appropriate way to collect and provide sufficient evidence that can be used to inform and impact health system policy decisions. Secondly, it is a means of obtaining a more complete in-depth understanding about diabetes as it is experienced and managed in urban and rural Cameroon. Thirdly, the comparative focus provides an overview of potential variations within a country like Cameroon. These settings, by their location and composition, seem different from each other, but to what extent is this the case?

### Ethical clearance, administrative clearance and informed consent

Ethical and administrative clearances were obtained from the National Ethical Committee of Cameroon and the Ministry of Scientific and Technical Research respectively. The Ministry of Scientific and Technical Research is the government authority that authorises scientific and technical research in Cameroon. These documents were used to further obtain authorisation and administrative clearance from the divisional officers, district medical officers and, in the Bafut case, of the traditional ruler, in order to get the local consent to conduct fieldwork.

The first contact in the clinic was with the diabetes clinic authorities to obtain their permission to recruit patients and use their clinics as observation sites. The participation of health care providers was also sought and since observation had to go beyond the clinics to the families, family consent was sought through the patients, then in an encounter with adult members of the family and heads of households. Patients whose families accepted participation in the fieldwork were included as part of the study and notification about the duration, frequency and the nature of the family visits and research were made. Wherever possible, a consent form was used and its purpose explained before conducting an interview or a focus group discussion. The participant(s), if literate, signed the consent form(s) and if illiterate gave a verbal consent.

### Data collection procedure

#### Design, sampling and recruitment

The participants in this study were people living with type II diabetes and obtaining care from health units. These people were married or widowed, and living within a family, to allow for the study of how families provided assistance to people living with diabetes in households and communities. The participants were required to be living with diabetes for at least one year. This was not a formal random sample; but the study did aim for coverage as representative as possible within the scope of a small-scale intensive study, on criteria of gender, age, and duration of diagnosed diabetes. A purposive 'sample' of patients was selected in both urban and rural settings with these criteria in mind.

Sampling in ethnography can be a bit of a misnomer [12]. The reality of daily life for an ethnographer is such that all contacts, all meetings, all events, and all associations are potential sources of insight and thus data. Because ethnography takes social relationships as its building blocks, ties and connections between people are automatically relevant, whether between 'sampled' individuals or not. In this fashion, fieldwork extended outwards from the 'sampled' clinics, patients and families to the wider community. For instance, in observing clinics, it would be misleading to suggest that fieldwork concentrated on sampled patients only. It was an opportunity to observe and mix with many of those attending diabetes clinics. The ethnographer who concentrates on an 'initial sample' would omit much relevant data. In that sense, the 'sample' most relevant for this study was the initial decision to select Biyem-Assi and Bafut as places to focus upon. Although patients were selected, this selection was most useful in providing a way to get started. One cannot say that health care providers or traditional healers were 'sampled', for these relationships developed more opportunistically. 'Snowball sampling' is the name sometimes given to a widening range of informants. But it is easy to use that term also to legitimate a series of events, contacts, encounters and methods that seem largely due to chance at the time, and in ethnography much is due to chance. The basic point is that the ethnographer should be 'there' in the first place, otherwise no relationships can develop.

Participants were recruited from four clinics (two from within each health districts). In the clinics, patients' consultations with health care providers were routinely observed including their reactions within the clinic milieu. Patients were selected in the clinics where they routinely consulted with a view to following their progress over the course of the year's fieldwork. This provided a list of twenty patients, therefore 20 families. A nurse opened access to patients during a diabetes clinic session, introducing the ethnographer as someone who was working on diabetes. Patients were initially informed that someone with a special interest in diabetes would be doing research with them. Fieldwork in the clinics got fitted around clinic appointment days and times, with non-clinic appointment days. The afternoons, evenings and weekends were the better times for visiting patients and their families at home. Once contact was made and some trust built up with patients, we met at clinic appointments, their homes and public places and ceremonies. In due course, as presence became more familiar, other patients asked to be visited. Visits were done to avoid the possibility that other people with diabetes attending these clinics might feel overlooked or given inferior treatment. These visits were often useful and served to extend the understanding of some hidden phenomena with regular participants. Each patient was visited four times in a week

Home visits extended to the wider community where the patients interacted with other structures of the society including those providing traditional medicine (traditional healers). Fieldwork covered a range of techniques, but aimed to relate what people say they did with what they said they ought to do and with what they actually did. For that reason, participant observation is the cornerstone of this study. In both settings, fieldwork took place for a year, a long enough period that one could be confident of witnessing situations which were largely unaffected by one's presence.

### Data collection tools

The different methods brought together within the overall framework of the participant observation included observation of and participation in public events and everyday situations, focus group discussions, in-depth interviews, fieldwork conversations, and case studies (biographical and event-based).

#### Observation

All observations were openly done, albeit discreetly where it was preferable not to draw endless attention to the researcher himself. Initially, only notes were taken; tape recorded information and pictures taken where people would not be suspicious. But over time, note taking became easier, especially when the observer was more visible and likely to be talked about. Because of the extended duration of fieldwork, people got used to and knew more about what the observer was doing. The observer tried to make sure that he was equally attentive to both ordinary and unusual situations, to record repetitive and more atypical events.

#### Interviews and Fieldwork conversations

An in-depth interview guide was used to follow through the interview and to cover themes that had emerged in the course of fieldwork. Interviews were tape-recorded wherever the subject accepted that. Moreover, a large number of fieldwork conversations with individuals or groups of participants were also taped, where that was possible. In both interviews and the more spontaneous fieldwork conversations, an informal style of questioning was adopted, with event and case driven questions preferred to make an easygoing dialogue possible. However, outside interviews, the observer equally refrained from asking questions but simply joined in conversations that people were holding. He could always raise an issue to have a point clarified or to elicit a response from a particular person. But the participant's point of view was respected. With time, themes and issues in conversations were identified, which were seen as important for exploring in subsequent fieldwork. It became easier to probe into certain topics where the observer was aware of gaps in fieldwork knowledge or in the published literature, or where there were conflicting viewpoints. Similarly, detailed biographies and case histories of my main informants were increasingly pieced together.

#### Focus group discussions (FGD)

Focus groups are a form of group discussions that generate data through the steered conversations of group members [14-16]. Although sometimes seen as a quick and convenient way to collect data from several people simultaneously, the group interaction aspect of focus groups also seeks to create by artificial means something of the conversational interaction, which any ethnographic study aspires to incorporate. This means that people were encouraged to talk to one another: asking questions, exchanging anecdotes and commenting on each others' experiences and points of view. The focus groups were linked to the overall process of continuous data collection, as the observer sought to make inroads into people's daily lives verifying issues that were raised in focus groups. The participants in focus groups were purposively selected. There were six participants in each focus group.

Focus groups were also chosen because they provided a way to help people explore and clarify their views in ways that might be less easily achieved in a one-to-one interview or even in one-to-one fieldwork conversations. Discussions were guided using a series of open-ended questions, and research participants were encouraged to explore the issues of importance to them, in their own vocabulary, generating their own further questions and interests in the topics raised, with a gentle steer from the researcher. Focus groups proved to be useful for studying dominant cultural values: for example, exposing influential narratives about lifestyle related to taking prescribed biomedical therapies and obesity or dealing with the stress of patients' illness management.

### Data management and analysis

The analysis was inductive and a continuous process from the early stages of fieldwork. It followed common convention for ensuring that the process was grounded in the data rather than reflecting a pre-determined analytic framework. In order to avoid forgetting vital details, note-taking and tape recording was done and transcripts produced almost always within 24 hours of observation. Conversational and content analyses of the data were made to produce the results here presented.

During fieldwork, analysis consisted of repeatedly reading transcripts and identifying the emerging themes for subsequent follow-up. New data helped to confirm or substantiate the integrity of the developing analysis; and an on-going dialogue with informants and study participants contributed to the shaping of themes and categories. The plausibility of the data interpretation was conducted to ensure that the qualitative data analysis was systematic and verifiable, as recommended by Dey [17] and Giacomini and Cook [18]. Analysis focused on both negative and positive outcomes aimed to find aspects related to compliance and non-compliance. This represented a stepping away from previous approaches, where only the negative aspects were considered.

The data collected for the study has been stored in an electronic database of the University of Newcastle as typed transcripts and can only be accessed by researchers using a personal password. The recorded tapes and notebooks have been safely stored in boxes where access is restricted to me alone.

## Results

The results are presented in five sections. We begin by illustrating the sociodemographic characteristics of patients on table 2. Subsequently, the results have been presented as: a) the meaning of compliance as a concept of dealing with competing regimes of treating diabetes; b) coming to terms with biomedical treatment of diabetes; c) the cost of diabetes as one of the triggers of non-compliance to biomedical regimes of diabetes; and d) the impact of AIDS in impeding adherence to some biomedical treatment of diabetes.

**Table 2:** Sociodemographic characteristics of diabetes patients observed for over a year.

Characteristics of participants	Urban	Rural
**Age groups**	n = 10	n = 10
35–50	4	1
51–65	3	3
>65	3	6
Median age	59	66
		
**Gender**		
Male	6	5
Female	4	5
		
**Marital status**		
Single	2	0
Married	8	9
Widow	0	1
		
**Occupation**		
Retired	3	4
Civil servants	5	2
Business	2	2
Farming	0	2

### Dealing with competing regimes of treatment for diabetes

Two competing regimes of treating diabetes emerged from the analysis: biomedical therapy on the one hand and traditional medicine on the other hand. Our data suggest that these two regimes are equally valued, contrary to the initial premise that people with diabetes would prefer biomedical therapy to any other alternative. The following remark makes this clear.

I think that biomedicine and traditional medicine are two sides of the same coin. They serve the same purpose by treating my diabetes. I cannot stop one to favour the other. It will mean that I am committing suicide.

(60-year old male diabetes patient in Yaounde)

The above quote raises the issue of compliance to treating diabetes as raised by health care providers, against the understanding of people with diabetes.

You prescribe medication to patients and they return worse than they were in the previous consultations. It means that they do not comply with treatment. I am convinced that they also treat themselves with traditional medicine.

(40-year old nurse in Yaounde)

People's understanding of treatment for diabetes and the concept of compliance are different from the prescribed definition of the term, hence provoking a tussle between biomedicine and traditional medicine as illustrated by the two quotes below:

Instead of going to purchase medicine in the clinics, it is better to harvest some leaves behind your house, boil and drink them like tea every morning. It will reduce the sugar in your blood to a normal level... Guava and mango leaves add insulin in your body. If you can heal on leaves, why go to the clinics to buy medicines ... ?

(73-year old Male diabetes patient in the rural health district).

... When you do not know what is happening to you, you will go anywhere to seek a cure. If the insulin in the clinic was effective, I will not be turning to traditional medicine. ... One alternates because one is desperate to obtain a cure for diabetes. ...

(54-year old female diabetes patient in the rural health district).

The two quotes above are extracts from a conversation with two people with diabetes. Both of the patients quoted have had diabetes for some time, and count as 'experienced' diabetes patients. This is an important distinction because the passage of time as a diabetes patient is also necessarily a phase of learning how to 'manage' the disease. In these extracts, there are several interesting points to note: the use of the word diabetes, and an understanding that diabetes cannot be cured. The use of the word diabetes is acknowledgement of the presence of a disease and not only an illness. These are coupled with recognition that people desire to believe that 'you can be cured' (second extract); and a demystification of the treatment offered by clinics and some traditional healers, suggesting that a person can just as easily treat himself (first extract). Such statements will arguably bring some qualified reassurance to the clinics, for they convey an acceptance of at least some of the lessons the clinics seek to put across. Yet, there is a gap that is apparent between clinic expectations of patient behaviour and the thinking and practice of patients themselves, and their families. Around the partly chronological series of aetiology and diagnosis, treatment and prognosis, there have emerged major contrasts between the world of the clinic and the world of patients. It is this divergence that heightens the clinic rhetoric of 'non-compliance', and which leads patients to be cast by clinics as deviant, to a greater or lesser extent.

However, from the perspective of many people with diabetes, there are other pressures to take into account and in a sense to conform to. The major pressure is the belief that illness is a form of misfortune.

Joan had been living with diabetes for 10 years and has not been able to obtain her expected cure, despite all attempts to be treated with biomedicine. She has not been able to get an explanation for her continuous ill health. She thought her colleagues had bewitched her. But at the request of her family, she consulted with a traditional healer. The traditional healer informed her that she has neglected her ancestors by not performing annual sacrifices to them, so they punished her with diabetes and even blocked the efficacy of any treatment provided to her at clinics. The traditional healer prepared some herbal tea for Ngon promising a cure if she performs the rituals and respected her ancestors in the subsequent years.

(Case study fieldnotes in Yaounde, 23 October 2001)

Joan had depended on biomedicine alone for treating diabetes with the expectation that she will obtain a cure, but this never happened. The inability of obtaining an explanation for her inability to cure her diabetes called for her to seek alternative explanations. Biomedical explanations can readily be fitted into Joan's frame of thinking up to a point. But biomedicine does not provide a sufficient explanatory framework for most Cameroonians and Africans alike, whether in rural areas or in the city. Ill-health is seen as having social causes, and 'complying' for most patients, if it has any meaning at all, means recognising precisely the social dimension to causation, where resolution is likely in the end to be found through the performance of rituals. Whether the road to ascertaining which rituals need performing runs via a traditional healer or not, only the restoration of compromised social relationships will create the possibility of dealing with diabetes properly and appropriately, which is to say for many attaining a 'cure'.

'Compliance' is a biomedical idiom underpinned by certain biomedical assumptions and values, which makes sense only to the health care provider. The other actors in the care process are largely unfamiliar with it, and rarely if ever employ it themselves. The accusation of being 'non-compliant' can easily sound senseless to patients, as they take it for granted that the causes of diabetes have a wider scope than biomedical health care providers understand. The following quote is illustrative.

I was never at ease when the doctor told me that I had diabetes. I tried to be cured in the hospital and my diabetes could not finish. The doctor kept telling me that I was not complying with treatment. A friend of mine also, sick with diabetes, told me that some unperformed ritual might be holding back the treatment. She advised me to consult a traditional healer. The healer advised me on the ritual to perform to my ancestors. After the ritual, I felt well and my blood sugar returned to normal six months afterwards. I believe that the doctor did not know this other cause of my diabetes and how I could be cured.

(60-year old male diabetes patient in the rural health district)

**Figure 1:**
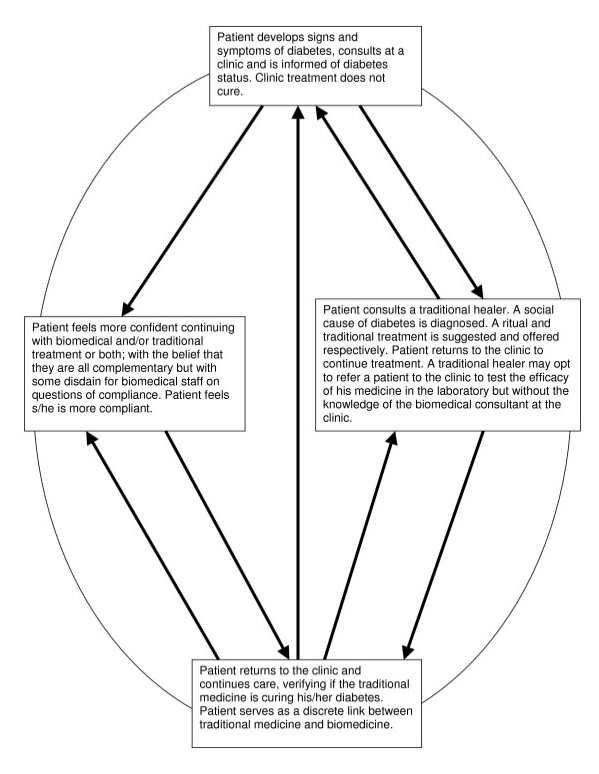
The healing cycle of diabetes.

In popular understanding, diabetes warrants treatment seen as potentially commensurate with its 'causes'. Many people with diabetes can easily think that their problems are only partially understood by the biomedical health care providers, so make recourse to traditional medicine to complete what the clinic cannot assist with, thus they hope and believe completing the healing cycle of diabetes as illustrated on Figure 1.

The behaviour of people with diabetes is evident in the treatment cycle (Fig 1) and in the statement commonly used while patients are in clinics, 'Let health care providers do their part, the traditional healers will complete the treatment'. They adopt other medical treatments in combination with biomedicine and believing that the combination will be more effective in curing diabetes.

I take both traditional medicine and biomedicine because it is more effective in curing me. But some patients take just one of them. It cannot be as effective as if you combined both.

(50-year old female urban diabetes patient)

From the biomedical perspective, such a rationale is nonsensical, and the patient is simply failing to comply as advised, and notably failing first by turning to traditional medicine, and second by ignoring the need to control blood glucose.

Doctor A narrated his frustrations of making extra efforts to enable his patient with diabetes comply to biomedical treatment and adopting lifestyle measures to prevent risk factors. He complained that despite all, many people with diabetes return to the clinic in conditions worst than the previous appointment because their blood sugar remains high.

(Extract from fieldnotes of an observation of a health care provider 24 January 2002)

### Coming to terms with biomedical treatment of diabetes

The ultimate aim of virtually all diabetes patients at the point of diagnosis – and indeed afterwards – in Cameroon is still to be cured, so that when one talks of treatment it is equated with curing and not management.

Every illness has a cure but my diabetes has not yet been cured despite all the medicines that I have taken. The doctor keeps reminding me that I will take medicines for life. Is there something particular with diabetes?

(65-year old female rural diabetes patient)

Such an argument, as with the quote, easily leads some people with diabetes to seek traditional medicine. Some people with diabetes even suggest that perhaps their mistake is to be combining too many treatments in an effort to find the cure.

May be my error is that I have been combining many medicines. I will try a few, say one or two, and see what difference it can make. If it is effective, I will stay with that.

(44-year old female rural diabetes patient)

So scepticism about the efficacy of biomedicine and what clinics can offer runs deep, as the following remarks convey:

... If scientists can discover a bark of tree, medicine to cure AIDS at the Korup National Park, what about diabetes? I am convinced that you have one there or in another forest that can cure diabetes...

(55-year old female Urban Diabetes patient).

Even many of the educated elite, who may on one level understand that diabetes cannot be cured, still think that a cure may be obtained through intensive research within the wide range of medicinal plants found within the Cameroonian flora.

I have the feeling that those conducting research on diabetes are not making enough efforts to identify and produce the medicines that can cure diabetes. Those researching on HIV/AIDS medicines are making advances. But researchers for diabetes medicines are unable to make similar advances.

(48-year old male urban diabetes patient)

Many educated people do not readily accept that other diseases may have a cure but diabetes is incurable and without a cure in prospect. However, there is more than scepticism about the efficacy of biomedicine even when complications set in.

... You go to the hospital, come back and after a few days you are fine. It comes back again. But the traditional healer will tell you the type of diabetes that can be treated in the hospital and the type that can be treated by him. When you disobey and go to the hospital you die, because they will just waste your time in the hospital...

(52-year male diabetes patient in the rural health district).

Slowly, a few Cameroonians are coming to terms with the fact that diabetes is not a curable disease.

I will stop alternating between traditional medicine and biomedicine and stick to biomedicine alone because even when you turn to traditional medicine they still send you back to consult your doctor at the clinic to verify if the traditional medicine is effective. They also send you to the clinic after their rituals, informing you that there is diabetes that can be cured and another that cannot be cured. It means that there is no cure or that something is holding back the cure.

(48-year old male urban diabetes patient)

Faced with the fact that cures do not happen (illustrated in quote), and aware of the chronic nature of the diabetes and diabetes complications, there is a tendency to explain such uncomfortable truths by arguing that there were delays in starting treatment, or that something is holding back the cure.

### Cost of biomedical treatment as a trigger to non-compliance

Financial considerations are also a major deterrent to following the biomedical path.

When prescriptions are made at the hospital, I have to wait for my children to buy them for me. This may take a few days to one week. It means staying without taking my drugs. But when I go to a traditional healer, he may ask just for a chicken and my herbal portion will be ready in the next one hour.

(64-year old rural female diabetes patient)

Many patients lack the money to purchase the medicines prescribed at diabetes clinics. Some patients think that these medicines are expensive and turn to traditional medicine with the belief that they are cheap but may end up paying more in kind. Again, the turning to traditional medicine may be relating to the patients' perceptions of social causation and their attitudes to traditional medicine. Most patients with these beliefs subscribe to healing rituals, which give them a feeling of relief.

The absence of health insurance policies has made it difficult for patients to obtain quality diabetes care, and many patients simply lack the money – or consider themselves as lacking the money – to go through with the full advice and treatment diabetes clinics propose. The very business of accessing the samples of patients through clinics initially might be said to skew this study towards those who at least had the funds to seek biomedical care. Some patients dip in and out of biomedical treatment regimes for precisely this reason. Even the prominent charitable clinics charge sums, which can bear very heavily on poor families, whatever the extent to which their costs are subsidised. It is in this context that traditional healers assume importance, for invariably their procedures are seen as much less costly and more culturally appropriate. The promise of being cured, and for far less cost, is a seductive one for those yet to be persuaded by the credibility of diabetes clinics. Healing based on local herbs, which may be abundantly available in the bushes in rural areas or cheap to buy from markets in towns, commands enormous support. At times, people may also conclude that the medicines prescribed at such a cost in the clinics are fabricated from these same readily available plants, reinforcing the state of mind that there are more viable and less ruinous ways to treat diabetes than by following every stipulation of clinic regimes.

### The impact of AIDS on accepting prescriptions

The emergence of diabetes as a significant public health problem across sub-Sahara Africa has coincided with HIV/AIDS. Both are chronic diseases, and neither can be cured. But for the diabetes patient, a major consideration is precisely not to be mistaken for an AIDS sufferer. The following quote illustrates.

... My weight was 90 kg when I was less than 35 years old. I have lost more than 10 kg as part of my treatment. People now perceive me as someone that is suffering from AIDS. This is affecting my personality. I have to gain some weight to remove that stigma and show them I am well...

(55-year old male urban diabetes patient).

From the quote, the impact of AIDS on certain wider health beliefs and practices should not be under-estimated. It is an issue of stigma attached to AIDS that over-spills to actions relating to diabetes treatment. In this possibility of confusion, weight is the key, as the quotes above shows. Sudden weight loss in Cameroon, as in most of sub-Saharan Africa, is now readily associated with having AIDS. Yet one of the main ways of managing diabetes is through weight control, which usually means weight loss. Advice to lose weight is something that most diabetes patients find instinctively difficult to follow, because of the fear of being thought of having AIDS. From the remark quoted, weight loss, although now overwhelmingly associated with popular attitudes to AIDS, actually goes beyond AIDS. For what is now being considered 'obesity' and 'overweight' has in the past and is still today taken as a sign of good health, wealth and vitality. While diabetes patients will often accept that weight loss can improve their quality of life and health, the very idea flies in the face of some deeply held assumptions about the ideal body. The ideal body being subsumed to one that is big.

## Discussion

This study reveals a complex web of understandings and interactions that influence compliant behaviour within a group of people with diabetes. Although all the findings are not new with respect to existing literature, they add substantially to what has been found previously, in other contexts. The study also provides context-based data about the interpretation of the value of clinical consultations for diabetes from the diabetes patients' perspective. At first glance, the results indicate negative feelings towards biomedicine, low awareness about biomedical therapy for diabetes and dissatisfaction with biomedical treatment for diabetes. In many respects, people with diabetes certainly saw themselves as seeking to follow clinic advice. However, the belief that patients were doing their best to follow these guidelines rested on the assumption that supplementing visits to the health care providers with visits to traditional healers was a sensible course of action, and very far from being deliberate or flagrant disregard of biomedicine. Patients, on the one hand, and health care providers, on the other, construe the same actions quite differently. Moreover, what health care providers express in terms of 'compliance' is commonly construed by patients in terms of restrictions and prohibitions, a long list of do's, don'ts, and costs. Traditional medicine escapes this image problem, working as it does with the grain of popular knowledge rather than in challenge to it.

Although compliance, adherence and concordance have been extensively researched, it has been argued that the outcome of much of this work provides little consistent information other than the fact that people do not always follow the doctors' orders [11,19-21]. The main function of such terms, according to some people with diabetes is ideologically to provide a framework for doctors to express their ideas about how people with diabetes ought to behave [7,22,23]. In recent years, the idea of *lay expertise *has been given some prominence, whereby people with diabetes gradually come to accept their diagnosis, and, gain mastery in coping with it [7,18,24-26]. It has been suggested that the psychological and physical stresses of diabetes call for specific adaptive and coping strategies, and that many people diagnosed with diabetes experience difficulty in adapting to biomedical therapy [5,27-29] and so shop for healers [30,31]. The social context of people with diabetes may have a profound impact on decisions to comply with and adhere to biomedical treatment. However, people with diabetes often identify a much broader set of constraints that influence treatment, including work, housing, finance, family and emotional factors. Dietary and medical non-compliance does not occur as a result of an idea or whim on the part of the patient (though of course it may) but, rather systematically, as part of competition between constraining factors.

Most existing literature about compliance concerns the richer countries of the world. Even so, there are parallels here with Cameroonian circumstances, in the sense that in Cameroon, as elsewhere in Sub-Sahara Africa [30], the language of compliance and adherence is the language of biomedicine. It is part of the modern repertoire of clinical surveillance, whether applied to low income countries of Africa or developed ones. Do Cameroonian patients absorb this language, and see their own behaviour and actions in terms of degrees of compliance? As the ethnography presented shows, it is only to a rather limited extent.

One outstanding finding in this paper is the deep-seated public belief that diabetes, like all other diseases, is potentially curable. A public health message that promises patients a lifetime of 'managing' diabetes, learning to adjust and live with the limitations it imposes on them, requiring that they follow stringent 'rules' of behaviour, in order to avoid an acute crisis and likely death, is not an appealing one. But how much is changing, and to what extent will the next generation of diabetes patients deal with these choices and pressures differently? How much will the growing appeal of science transform those attitudes and frames of mind that have been discussed here? Are there clues in this regard if we contrast towns and villages? That is hard to say at this point. What we can say is that villages and towns, as illustrated by these findings, resemble each other more than they diverge. There is not to any marked extent, a rural analysis to present and a separate urban one. But perhaps the primary point is how resilient so-called 'traditional' ideas prove to be in relation to critical matters of health in the urban context.

### Limitations

The main limitation of this study lies in the relatively small sample of people studied. That is counterbalanced by the wealth of contextual data through which it has been possible to examine relationships and dynamics, which would have been beyond the reach of a large-scale survey. The element of bias in this study by virtue of working solely with those patients diagnosed previously, as diabetic, has to be acknowledged. It cannot be completely set aside in this fieldwork given that the researcher was dealing with patients known to diabetes clinics. The undiagnosed (yet to be or never to be diagnosed) cases clearly could not be part of this fieldwork. But the same would be true of any similar study of people suffering from a chronic disease: it is by definition impossible to define a 'community' of sufferers of an illness, disease or condition of which these individuals are unaware.

## Conclusion

The differences in the types of knowledge upon which people living with diabetes base their assessments can lead to disagreement and mutual resentment in which health care providers accuse them of not grasping the severity and implications of their condition, and its life-threatening complications. Generally, people with diabetes consider such charges as unjustified. This, in essence, is the discourse and conflict around compliance. But the task of this paper has been to show how people with diabetes make sense of their circumstances. This means showing why what clinics regard as 'non-compliance' is seen very differently from the patients' side. This ethnography shows how different individuals seek different paths of treatment for themselves, some being much more convinced than others do by the efficacy of biomedicine. However, there is a need for broad-based health education and health promotion for people with diabetes, health care providers (biomedical and traditional healers) and families about diabetes, with apparent need for health insurance schemes.

An exploration of these issues may help the physician discuss with the patient the appropriateness of the proposed treatment and to more openly discuss the limitations of alternative kinds of therapy, rather than making it a taboo subject that the patient dares not raise for fear of a withering response. This implies that a new perspective on health care, one which goes beyond a flat assertion of biomedicine's therapeutic hegemony, is needed and that health care providers must become more active in their interactions with patients and be able to examine the patient's understanding of the world of disease and illness. This will move clinical interactions in Africa from the premises of compliance towards the spirit implied in notions of adherence and concordance. To achieve this goal, health care providers should be trained in interpersonal skills. This will form part of the efforts in building strong health care systems in Africa.

## Abbreviations

AIDS: Acquired Immune Deficiency Syndrome; ANSA: Action on Noncommunicable Diseases in Sub-Sahara Africa; FGD: Focus Group Discussion; HIV: human immunodeficiency virus.

## Competing interests

The author(s) declare that they have no competing interests.

## Authors' contributions

PKA conducted the ethnography fieldwork on diabetes for a PhD thesis with the University of Newcastle upon Tyne, United Kingdom. PRP and NCU of the University of Newcastle upon Tyne supervised the thesis. This ethnography has provided data for this analysis. We certify that we conceived and designed this work and analysed the data, as well as in the writing of the manuscript to take public responsibility for it. We believe the manuscript represents valid work. We have reviewed the final version of the submitted manuscript and approve it for publication bearing our names. Neither this manuscript nor one with substantially similar content under our authorship has been published or is being considered for publication.

## Pre-publication history

The pre-publication history for this paper can be accessed here:


